# Hepatocellular Carcinoma Immunotherapy: Predictors of Response, Issues, and Challenges

**DOI:** 10.3390/ijms252011091

**Published:** 2024-10-15

**Authors:** Alessandro Rizzo, Oronzo Brunetti, Giovanni Brandi

**Affiliations:** 1 S.S.D. C.O.r.O. Bed Management Presa in Carico, TDM, IRCCS Istituto Tumori “Giovanni Paolo II”, Viale Orazio Flacco 65, 70124 Bari, Italy; dr.oronzo.brunetti1983@gmail.com; 2 Department of Specialized, Experimental and Diagnostic Medicine, University of Bologna, Via Giuseppe Massarenti, 9, 40138 Bologna, Italy; 3 Division of Medical Oncology, IRCCS Azienda Ospedaliero-Universitaria di Bologna, Via Albertoni, 15, 40138 Bologna, Italy

**Keywords:** hepatocellular carcinoma, liver cancer, immunotherapy, immune checkpoint inhibitors, PD-L1, cancer

## Abstract

Immune checkpoint inhibitors (ICIs), such as durvalumab, tremelimumab, and atezolizumab, have emerged as a significant therapeutic option for the treatment of hepatocellular carcinoma (HCC). In fact, the efficacy of ICIs as single agents or as part of combination therapies has been demonstrated in practice-changing phase III clinical trials. However, ICIs confront several difficulties, including the lack of predictive biomarkers, primary and secondary drug resistance, and treatment-related side effects. Herein, we provide an overview of current issues and future challenges in this setting.

## 1. Introduction

Hepatocellular carcinoma (HCC), intrahepatic cholangiocarcinoma (iCCA), mixed HCC–iCCA, and other forms of primary liver cancer (PLC) are among the sixth most common cancers and the third cause of cancer-related deaths globally [1,2,3]. Among PLCs, HCC is the most common type, accounting for approximately 75–80% of cases [4,5,6]. Most HCC patients are diagnosed when the disease is in an advanced stage, and less than 20% of cases survive for five years [7,8,9]. Cancer immunotherapy includes several different therapeutic strategies, ranging from adoptive immunotherapy to cancer vaccines and immune checkpoint inhibitors (ICIs) [10,11,12,13,14,15,16,17], and the use of several ICIs, either singly or in combination, has increased the efficacy of HCC systemic treatment, although there are still many issues to be faced [18,19,20].

ICIs, such as cytotoxic T-lymphocyte antigen 4 (CTLA-4) inhibitors, programmed death ligand-1 (PD-L1) inhibitors, and programmed death-1 (PD-1) inhibitors are frequently used, as monotherapy or in combination—such as durvalumab plus tremelimumab and atezolizumab plus bevacizumab [21]. From a biological point of view, PD-L1 binds PD-1 and can inhibit T-cell proliferation and cytokine secretion function. The co-stimulatory molecule PD-1, also referred to as CD279, belongs to the CD28 family and is expressed on the surface of T cells, B cells, and natural killer (NK) cells [22]. Two B7 family members, PD-L1 and programmed death ligand-2 (PD-L2), are ligands for PD-1, and while PD-L2 is primarily expressed on macrophages and dendritic cells (DCs), PD-L1 is expressed on the surface of tumor cells as well as on certain immune cells like macrophages, T cells, and DCs [23,24]. In a physiological setting, PD-1 and PD-L1 together can protect the body’s immune balance, prevent excessive immune responses, and maintain autoimmune tolerance by sending inhibitory signals to activated T cells [25,26]. Nonetheless, through the expression of high levels of PD-L1 and its combination with PD-1 on the surface of T cells, tumor cells may prevent T cell activation and trigger tumor cell immune escape [27,28]. Exosomal PD-L1 is a substance that tumor cells have been demonstrated to secrete to suppress T cell activity [29]. Owing to the critical roles that PD-1 and PD-L2 play in tumor immune escape, antibodies that block this pathway restore T cells’ ability to kill tumor cells through immune-killing [30]. Another significant ICI with a strong binding affinity for the B7 family of co-stimulatory receptors is CTLA-4. Consequently, CTLA-4 inhibits second co-stimulatory T cell signaling by binding to these receptors more easily than CD28 [31]. Normal circumstances allow CTLA-4 to inhibit non-essential T cell activation, which in turn regulates hyperactive T cell immune responses. On the other hand, increased CTLA-4 expression in tumors prevents T cell activation, proliferation, and effector function [32]. As a result, targeting CTLA-4 and utilizing T cells’ antitumor killing ability has emerged as a successful tumor treatment strategy.

Based on these premises, this review explores current challenges of HCC immunotherapy, discussing the role of emerging biomarkers and mechanisms of resistance and toxicity in ICIs clinical trials.

## 2. The Immunological Milieu of Hepatocellular Carcinoma

**Figure 1:**
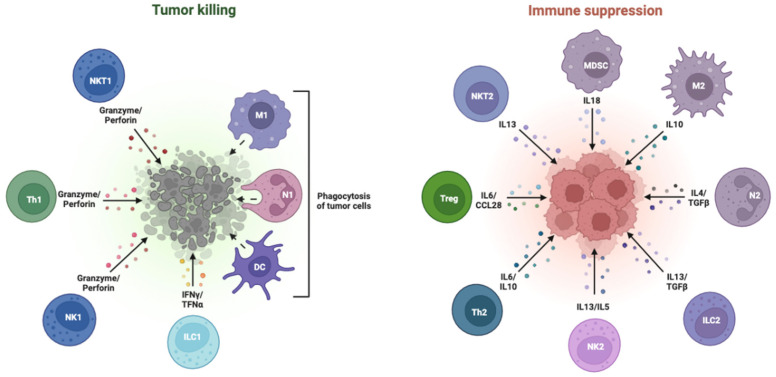
Pro- and anti-tumor cells in the tumor microenvironment.

As a key organ involved in immune regulation, the liver maintains a dynamic balance between inducing immune tolerance to prevent immune damage and triggering an immune response to eliminate antigens [33]. This property protects against the potential harm caused by autoimmunity and chronic inflammation under normal circumstances. Nevertheless, this process results in immune escape from the tumor and impairs the immune response to tumor antigens [34]. Owing to the physiological features of HCC, tumor, immune, and stromal cells form a unique tumor microenvironment (TME) in the liver (Figure 1) [35].

In addition to stimulating the growth of tumor cells, TME has several important immune-suppressive effects, including preventing immune cells from activating, killing, or slowing down immune cell division, and encouraging the development of regulatory T cells (Treg) [36]. The TME of HCC involves several cells: hepatic macrophages known as Kupffer cells play a crucial scavenger role in the innate immune system and create an immunosuppressive milieu and induce immune tolerance [37]. Kupffer cells in HCC overexpress PD-L1, which attaches to PD-1 on CD8+ T cells to prevent T cells from killing other T cells. In the meantime, Treg cells in HCC can secrete inhibitory cytokines, cause effector lymphocytes to undergo apoptosis, and impair the activity of DCs [38]. Furthermore, several signaling pathways exist within the TME. For example, cell cycle-associated kinases can trigger the hepatic EZH2-NF-kB pathway, which in turn promotes the development of HCC and immune escape by accumulating myeloid-derived suppressor cells (MDSCs) [39,40]. Consequently, the unique physio-pathological traits of HCC make treatments more challenging.

## 3. Biomarkers of Response to Hepatocellular Carcinoma Immunotherapy

**Figure 2:**
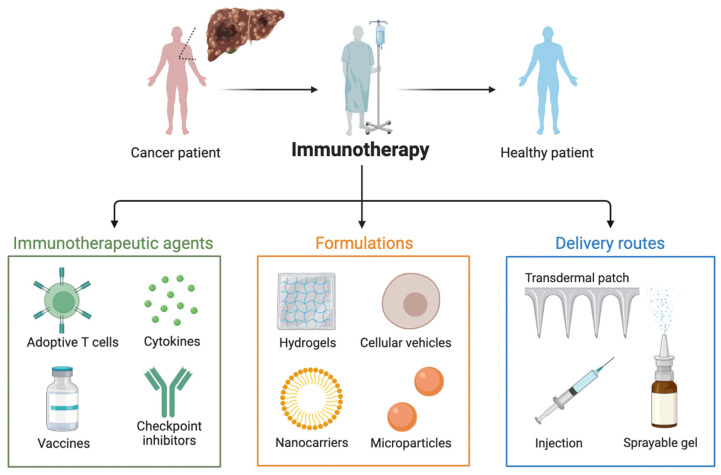
Immunotherapies in hepatocellular carcinoma patients.

The development of ICIs has completely changed the treatment landscape for HCC, improving the efficacy of systemic treatments and extending overall survival (Figure 2) [41,42,43]. However, many HCC patients do not get any benefit from ICIs such as durvalumab plus tremelimumab and atezolizumab, and finding reliable predictive biomarkers to screen the most suitable candidates to ICIs and increase the effectiveness of immunotherapy represents an important clinical and research task [44,45,46].

According to mounting evidence, patients with high expression of PD-L1 are more likely to respond better and longer to ICIs, since PD-L1 is considered an important target for ICIs and a marker for predicting the efficacy of immunotherapy and immune-based combinations. In the KEYNOTE-224 trial, the investigators evaluated PD-L1 expression by utilizing the combined positive score (CPS) and tumor proportion score (TPS) [47,48,49]. According to the results of this study, TPS did not correlate with patients’ response to the PD-1 inhibitor pembrolizumab, whereas CPS did. In CheckMate-040, the authors observed that the effectiveness of nivolumab was unrelated to TPS; however, median overall survival (OS) of patients with PD-L1 ≥1% and <1% was 28.1 and 16.6 months, respectively (*p* = 0.03), in a later analysis exploring the relationship between PD-L1 expression in tumor tissue and OS in the CheckMate-040 study [50,51,52]. At the same time, it is worth noting that the study was retrospective, and the sample size was relatively small; to validate these findings, more information is required. Among the several variables that have been suggested to affect PD-L1 as a biomarker for ICIs, the heterogeneity of PD-L1 expression itself is an issue. Pinato and colleagues highlighted a significant heterogeneity in the expression of PD-L1 in tumor tissues, immune cells infiltrating the tumor, and non-tumor cirrhotic tissues [53]. This heterogeneity may have an impact and play a role on the accuracy and consistency of PD-L1 as a predictor of the effectiveness of ICIs. The positive expression of PD-L1 ranged from 2% to 10% in HCC tissues, from 6% to 22% in tumor-infiltrating immune cells, and from 2% to 19% in non-tumor cirrhotic tissues, according to the authors’ analysis of 100 HCC specimens from three centers. Meanwhile, different ICIs are based on different antibodies because of variations in detection platforms, and there is no common evaluation standard. Of note, PD-L1 expression is a dynamic process, while PD-L1 is typically assessed at a single moment in time, and thus, more research is needed in this setting.

The most assessed serologic marker of HCC, α-Fetoprotein (AFP), is highly expressed in about 70% of patients and is used for prognostic evaluations in addition to HCC screening and diagnosis. Patients with pretreatment AFP levels higher than 20 ng/mL had their response assessed in a retrospective analysis involving 60 advanced HCC patients receiving ICIs [54]. Within the first four weeks of starting treatment, a patient’s AFP level should drop by more than 20% in comparison of starting treatment; of these patients, 43 could be evaluated for early AFP response, and these findings indicated that early AFP responders had a longer median OS (28.0 months vs. 11.2 months, *p* = 0.048) and median progression-free survival (PFS) (15.2 months vs. 2.7 months, *p* = 0.002), as well as higher overall response rate (ORR) (73% vs. 14%, *p* < 0.001). In another retrospective study, researchers observed that AFP reduction >10% (within 4 weeks) was an independent predictor of ORR (OR = 7.259, *p* = 0.001) [55]. When comparing patients with baseline AFP ≥ 10 ng/mL, those with early AFP reduction had significantly higher ORR (63.6% vs. 10.2%, *p* < 0.001) and disease control rate (DCR) (81.8% vs. 14.3%, *p* < 0.001) than those without it. These findings imply that improved ICI outcomes could be linked to an early AFP response [54,55]. More research is required to validate these studies, as they are retrospective and have limited strength and quality of evidence. However, there are certain patients who do not have elevated AFP, and using this indicator as a biomarker has some limitations because it cannot be evaluated prior to treatment and must be applied based on post-treatment data.

Tumor-infiltrating lymphocytes (TILs) include cells with immunosuppressive activity and cells with anticancer activity, and are intimately linked to the effectiveness of immune checkpoint inhibitors [56,57,58]. In fresh and archived tumor tissues from the CheckMate-040 study, the relationship between CD3+ T cells and CD8+ T cells on OS was explored [52]. There was a trend towards longer OS, despite the fact that the increase in CD3+ and CD8+ T cells was not statistically significant (*p* = 0.08 and *p* = 0.08, respectively) in relation to OS. More recently, a noteworthy rise in CD8+ T cells was noted in the clinically beneficial population in a study assessing tremelimumab in combination with tumor ablation for the treatment of HCC. More thorough research is necessary to determine whether TIL is a useful biomarker for HCC.

About one-third of patients with HCC have activating mutations in *CTNNB1*, a gene that encourages immune evasion and ICI resistance. The WNT/β-catenin pathway, which is activated by mutations in the *CTNNB1* gene, was found to be associated with a lower disease control rate (DCR) (0% vs. 53%), a shorter median PFS (2.0 months vs. 7.4 months), and lower median OS in 31 patients treated with ICIs, as compared to WNT wild-type (9.1 months vs. 15.2 months) [59,60,61]. Consequently, *CTNNB1* is under evaluation as a potentially useful biomarker to guide the use of ICIs in HCC.

Tumor mutational burden (TMB) is a significant independent indicator of the effectiveness of ICIs [62,63]. According to multiple studies, patients who have more TMB (greater than 10 mutations per million bases) after receiving ICIs have better response rates and longer survival times [64]. The KEYNOTE-158 study assessed the relationship between TMB and prognosis in patients receiving pembrolizumab treatment for advanced solid tumors [65,66,67]. The study examined 790 patients whose TMB status could be determined; 102 (13%) of these patients had a high expression status of tissue TMB (tTMB), and 688 (87%) patients did not. As compared to the non-tTMB high expression group, which had an ORR of 6% (95% confidence interval, CI: 5–8%), the results of the tTMB high expression group showed an ORR of 29% (95% CI: 21–39%), indicating a stronger tumor response to pembrolizumab. However, Yarchoan and colleagues suggested that HCC had a low number of somatic mutations encoded per megabase [68]. In this study, only six tumors (0.8%) out of 755 patients with unresectable HCC that underwent whole genome sequencing analysis had high TMB. Furthermore, to date, no clinical trial has confirmed that using TMB to evaluate the effectiveness of ICIs in HCC is effective. The predictive value of TMB in HCC should be further investigated, however, as it has demonstrated strong predictive qualities in other tumor types. Another frequently used biomarker to evaluate the efficacy of ICIs is mismatch repair (MMR) status [69,70,71]; for example, a phase II study evaluated the response of colorectal cancer to pembrolizumab, with or without MMR deficiency (dMMR) [72]. The ORR and PFS rates with dMMR were 40% and 78%, respectively, while the rates without dMMR were 0% and 11%, respectively. However, few data are available regarding HCC, and dMMR is only present in 2 to 3% of all cases [73].

The microbes known as gut microbiota are long-term residents of the digestive system that interact with the human body and have a significant effect on tumors and anticancer treatments [74,75,76]. According to recent research, the gut microbiota may play a major role in modifying the effectiveness of ICIs [77,78,79]. In a report published by Vétizou and colleagues, ICIs were ineffective in mice receiving antibiotic treatment or kept in an aseptic environment [80]. However, the efficacy of immunotherapy was higher when gut microbiota was added, something that suggested that the microbiome could play a key role in modifying the response to ICIs. In particular, fecal samples from ICI responders may exhibit greater counts of genes and higher taxa than non-responders, according to a study by Zheng et al. [81]. In fact, the gut microbiota of ICI responders and non-responders changed dramatically and showed notable differences during immunotherapy. The effectiveness itself of ICIs may be predicted by examining these variations in gut microbiota, making the microbiome a potential biomarker as well. Given the extreme heterogeneity of HCC, using a single biomarker to direct systemic treatments may not be appropriate [44,82,83,84]; instead, combining multiple biomarkers could represent a more beneficial strategy. In addition, the fact that entirely different outcomes were obtained for subgroup analysis due to the use of distinct biomarkers highlights the significance of tumor biomarkers in drug use. Lastly, the study of biomarkers for HCC immunotherapy would need the further development of high-throughput sequencing, gene editing, and artificial intelligence technologies.

## 4. Current and Future Challenges

The introduction of ICIs has marked a fundamental moment in the treatment scenario for advanced HCC [85]. However, several issues still need to be faced, including—as previously stated—the identification of reliable predictive biomarkers, the safety profile, and drug resistance to ICIs and immune-based combinations. Resolving these issues is crucial to enhance the efficacy of HCC immunotherapy.

Drug resistance is due to several mechanisms in HCC. Tumor antigens are produced in low quantities in this liver tumor, and HCC is a disease whose somatic cells typically contain few mutations [86]. Moreover, liver has an extremely immune-tolerant environment, and loss of signaling pathways also contributes to HCC drug resistance. For example, deletion of *PTEN* reduces T-cell translocation to the tumor and inhibits T-cell-mediated tumor killing, while activation of the beta-catenin signaling pathway increases tumor immune escape and resistance to ICIs and immune-based combinations [87]. Taken all together, these factors are significant contributors to ICI primary resistance. Conversely, when an immunotherapeutic agent is effective at first but eventually loses its effectiveness, this is referred to as acquired resistance [88,89]. The two main causes of this are T cell depletion and the lack of neoantigen production. T cells are crucial immune cells that fight tumors, but as tumors grow larger or as long-term immunotherapy is administered, T cells gradually lose some of their anticancer properties and several “negative” immune checkpoints, including PD-1, TIM-3, LAG-3, and others, start to appear [90,91]. Another major factor contributing to acquired resistance is the decreased production of tumor neoantigens [92,93]. Following the administration of ICIs, clonal selection takes place within the tumor, allowing cells with low immunogenicity or low neoantigen expression levels to survive and complicating the induction of antitumor immunity in vivo.

Moreover, novel resistance mechanisms are constantly being discovered as research advances. For example, a recent study published by Tan and colleagues highlighted an isoform of PD-1 known as Δ42PD-1 [94]. In this report, the authors observed that up to 71% of untreated HCC patients expressed Δ42PD-1, which was correlated with a more aggressive clinical course. Compared to PD-1-positive cells, the study reported that Δ42PD-1-positive T cells had a greater deficit in antitumor function. Patients with HCC who received ICI treatment demonstrated effective PD-1 treatment; however, over time, and especially in those whose disease progressed, the frequency of Δ42PD-1-positive T cells rose. The authors also showed that Δ42PD-1 blockade enhanced intratumor T cell antitumor killing activity and suppressed tumor growth in murine models. This research not only suggested some mechanisms behind the ineffectiveness of ICIs in some patients, but it also revealed Δ42PD-1 as a new target for HCC immunotherapy. Several strategies have been tested and are under assessment to overcome drug resistance in HCC immunotherapy [95,96]. For instance, combining radiation and systemic treatments can increase the presentation function of DCs, stimulate the ICD response, and encourage the expression of tumor antigens [97,98,99,100]. Additionally, the combination of anti-angiogenic medications can enhance drug distribution and encourage T-cell penetration [100,101,102,103].

Another key issue in HCC immunotherapy is the presence of immune-related side effects [104,105,106,107,108,109]. As a result, treatment-related adverse events (TRAEs) play a crucial role in the pharmacologic management of these medications. According to available evidence, TRAEs are reported in over 90% of HCC patients receiving anti-CTLA-4 antibodies and 70% of patients treated with PD-1 and PD-L1 inhibitors, with the most common types being gastrointestinal, endocrine, and cutaneous [110,111,112,113]. The majority of TRAEs are less than or equal to grade 3, and they can be better managed with appropriate care. However, certain toxic reactions, including myocarditis, pneumonia, and neurotoxicity, are rare but dangerous and even life-threatening. Combination therapy with ICIs is currently gaining traction as a treatment strategy for HCC, but it also coincides with an increase in the incidence of TRAEs [114]. According to recent evidence, more than half of patients receiving durvalumab plus tremelimumab may develop grade 3–4 TRAEs, compared to 37.1% of patients in the durvalumab monotherapy group. However, when compared to other tumors, the incidence of TRAEs in HCC patients is not significantly different, and these TRAEs can be successfully treated [115]. Meanwhile, liver dysfunction brought on by cirrhosis as well as extrahepatic manifestations may coincide with the symptoms due to TRAEs, since many HCCs are typically linked to a history of cirrhosis. Consequently, it is important to distinguish between them by looking at the patient’s medical history, symptoms, and signs.

If randomized controlled trials are widely accepted by researchers and clinicians to explore the safety and efficacy of anticancer drugs, the inclusion and exclusion criteria of these studies are extremely stringent and do not accurately represent the complex reality of clinical practice. Thus, to manage potential adverse reactions and to guarantee the efficacy of HCC immunotherapy, it is imperative to pay close attention to the underlying conditions of patients in the real world.

## 5. Conclusions

ICIs, as monotherapy or in combination with other anticancer agents, have gained importance in the treatment of HCC. However, the efficacy of ICIs in the general population is still insufficient, so efforts should be directed toward overcoming ICI resistance and addressing several issues, including TRAEs. Choosing the best course of action for each patient is a crucial aspect of treating HCC because patients differ in their physio-pathological conditions, tolerance, and response to various medications. To improve clinical outcomes and survival for HCC patients, it is critical to identify the most suitable biomarkers for the use of ICIs, to efficiently manage TRAEs, and implement tailored interventions for individual patients.

## Data Availability

Not applicable.
